# Type I error control in biomarker-stratified clinical trials

**DOI:** 10.1186/1745-6215-16-S2-O84

**Published:** 2015-11-16

**Authors:** Deepak Parashar, Jack Bowden, Colin Starr, Lorenz Wernisch, Adrian Mander

**Affiliations:** 1 University of Warwick, Coventry, UK; 2 MRC Biostatistics Unit, Cambridge, UK

## 

Biomarker-stratified clinical trials assess the biomarker signature of subjects and split them into subgroups so that treatment is of benefit to those who are likely to respond. Since multiple hypotheses are tested, it becomes important to control the type I error. Current methods control the false positive rate where one rejects the null hypothesis while in reality that was true. For two subgroups, the false positive rate is controlled across the two hypotheses as a Family Wise Error Rate (FWER) to an overall predetermined significance level.

The concept of “Wrong Positive” rate is presented where one rejects the null hypothesis in one subgroup while in reality the efficacy would be in the other subgroup. Controlling the wrong positives as well as false positives to an overall significance level constitutes a strong FWER. Further options exist for control of Individual Outcome FWER by assigning weighting to reject the respective null hypotheses.

We apply our methodology to an optimal biomarker-stratified Simon two-stage cancer clinical trial design and illustrate the variation in minimum expected sample sizes for different choices of type I controls for a range of operating characteristics. While the weak FWER would suit a phase II trial, additionally controlling the wrong positives could be more appropriate in a confirmatory setting. The chosen design is an adaptive enrichment that evaluates efficacy in biomarker-positive as well as biomarker-negative subpopulations, and optimal designs are obtained using the four different choices of type I error controls: weak or strong FWER with or without individual outcomes.

